# Re: “Management of Suspected Bladder Injury and Capsular Perforation After Holmium Laser Enucleation of the Prostate” by Lwin et al. *(J Endourol Case Rep 2018;4:87–90)*

**DOI:** 10.1089/cren.2018.0074

**Published:** 2018-09-20

**Authors:** Jack Stutz, Colin Kleinguetl, Marawan El Tayeb

**Affiliations:** ^1^ Baylor University, Waco, Texas.; ^2^ Department of Urology, Scott and White Medical Center Temple, Temple, Texas.

Holmium laser enucleation of the prostate (HoLEP) is a well-established treatment option for benign prostatic hyperplasia (BPH) that has been gaining popularity in the United States. HoLEP is a minimally invasive procedure that involves enucleation of the prostate with subsequent morcellation and serves as an alternative to transurethral resection of the prostate (TURP), which is still considered the gold standard of treatment in many institutions and practices.

The literature demonstrates that HoLEP provides improvement of International Prostate Symptom Score, quality of life, flow (Qmax), and post-void residuals.^1^ It has also shown to have decreased or at least similar complication rates, such as bleeding and need for catheterization, compared with a TURP.^1,2^ In addition, HoLEP allows for the opportunity of increased removal of prostatic tissue. These findings have made the procedure increasingly more popular and favorable. However, HoLEP is well known for having a steeper learning curve and initial increased cost of materials compared with a standard TURP.^3^

This recent report describes the inherent risks of morcellation during HoLEP procedures with multiple case reports.^4^ The purpose of this editorial is to demonstrate the relative safety of morcellation during HoLEP.

In the first case, the authors report prolonged morcellation time and concern for bladder injury because of poor observation associated with bleeding at the bladder neck.^4^ Although exact details are unclear from the report, it needs to be emphasized that a clear endoscopic view is the most important aspect for safe morcellation. The first step of morcellation is to ensure the bladder is appropriately distended to ensure appropriate observation and to decrease risk of bladder injury. To achieve this, there should be a well-trained OR staff to monitor irrigation as well as maintaining two open inflow ports. This is always needed to assist with identification of the previously enucleated prostatic tissue and maintain bladder distention.^5^

One of the benefits of Holmium lasers is the relative ease of achieving hemostasis to greatly reduce the amount of bleeding during a procedure and the laser has known tissue penetration of about 5 mm. Maintaining hemostasis throughout the entirety of morcellation is critical. Bleeding must be controlled meticulously to ensure a clear endoscopic view before morcellation.^6^ Unfortunately, this crucial step appears to have been performed inadequately based on the description from the report. It is also important to maintain a minimal distance between the endoscope and the morcellator to improve precision and accuracy of morcellation, which, again, will decrease risk of bladder injury.^6^

As long as adequate hemostasis is achieved and a clear view of the adenoma and bladder is maintained, the incidence of injury should be minimal. Guidelines for safe morcellation should always be adhered to. If there is any question with regard to observation or excessive bleeding, morcellation should be halted immediately in favor of achieving better hemostasis and reidentification of crucial landmark. Failure to follow set standards puts the patient at increased risk for injury.

It is also crucial to keep the morcellator away from the bladder mucosa at all times, the morcellator should be kept in the middle of the bladder all the time. A specific technique has been illustrated that involves a continuous right to left motion of the morcellator while retrieving the tissue while gently advancing the morcellator in small increments and occasionally rotating it as necessary to optimize the engagement of tissue.^7^ It is important to understand that the retrieval of tissue should be performed in a manner such that there is no strain on the urethra and bladder neck with the camera/morcellator. If the enucleated tissue proves to be too large or there is any difficulty with morcellation, it is once again recommended that morcellation be stopped immediately until the tissue is carefully manipulated to optimize conditions before proceeding and always have open blades covering the prostate before morcellating. Forced tissue removal under suboptimal conditions will certainly risk avoidable injury.

HoLEP has proven to be a powerful technique in treating BPH. Its advantages over a standard TURP procedure have been well described in the literature. Proper safety standards exist to prevent bladder injury. In particular, maintaining observation with constant irrigation, achieving adequate hemostasis, and maintaining a safe minimal distance between the camera and morcellator are all necessary to reduce risk of complication, and retrieval of tissue should be performed in a manner to minimize strain on the urethra, bladder neck, and bladder itself. Although this report illustrates the inherent risks of HoLEP, specifically morcellation, the information provided suggests that standard safety techniques were not adequately followed. When performed appropriately, morcellation is a relatively safe procedure after enucleation of the prostate.

## Abbreviations Used

BPH: benign prostatic hyperplasia
HoLEP: holmium laser enucleation of the prostate
TURP: transurethral resection of the prostate

## References

[1] Alkan I, Ozveri H, Akin Y, Ipekci T, Alican Y Holmium laser enucleation of the prostate: Surgical, functional, and quality-of-life outcomes upon extended follow-up. Int Braz J Urol 2016;42:293–301 27256184 10.1590/S1677-5538.IBJU.2014.0561 PMC4871390

[2] Ahyai SA, Gilling P, Kaplan SA, Kuntz RM, Madersbacher S, Montorsi F, Speakman MJ, Stief CG Meta-analysis of functional outcomes and complications following transurethral procedures for lower urinary tract symptoms resulting from benign prostatic enlargement. Eur Urol 2010;58:384–397 20825758 10.1016/j.eururo.2010.06.005

[3] Baron M, Nouhaud FX, Delcourt C, Grise P, Pfister C, Cornu JN, Sibert L HoLEP learning curve: Toward a standardised formation and a team strategy. Prog Urol 2016;26:492–499 27614386 10.1016/j.purol.2016.08.002

[4] Lwin A, Hynes K, Tzou D, Funk J Management of suspected bladder injury and capsular perforation after holmium laser enucleation of the prostate. J Endourol Case Rep 2018;4:87–90 29938230 10.1089/cren.2018.0021 PMC6014571

[5] Kelly DC, Das A Holmium laser enucleation of the prostate technique for benign prostatic hyperplasia. Can J Urol 2012; 19:6131–6134 22316518

[6] Kim M, Lee HE, Oh SJ Technical aspects of holmium laser enucleation of the prostate for benign prostatic hyperplasia. Korean J Urol 2013; 54:570–579 24044089 10.4111/kju.2013.54.9.570 PMC3773585

[7] Kuo RL, Paterson RF, Kim SC, Siqueira TM, Elhilali MM, Lingeman JE Holmium laser enucleation of the prostate (HoLEP): A technical update. World J Surg Oncol 2003;1:6 12818001 10.1186/1477-7819-1-6 PMC165416

